# Precision Medicine in the Treatment of Locally Advanced or Metastatic Urothelial Cancer: New Molecular Targets and Pharmacological Therapies

**DOI:** 10.3390/cancers14205167

**Published:** 2022-10-21

**Authors:** Antonio Vitiello, Francesco Ferrara, Ruggero Lasala, Andrea Zovi

**Affiliations:** 1 Ministry of Health, Viale Giorgio Ribotta 5, 00144 Rome, Italy; 2 Pharmaceutical Department, Local Health Unit Napoli 3 Sud, Dell’amicizia Street 22, 80035 Nola, Italy; 3 Local Health Unit Bari, Hospital Pharmacy of Corato, Polytechnic University of Bari, 70100 Bari, Italy

**Keywords:** cancer, urothelial, therapeutic, molecular targets, precision medicine

## Abstract

**Simple Summary:**

Therapeutic breakthroughs in urothelial carcinoma have been occurring rapidly over the past 10 years. However, the resistance and prognosis of this disease is always quite a difficult health challenge. Trying to understand the new therapeutic perspectives is a priority to try to bring in new drug models that can counteract the advancing prognosis and inauspicious diagnosis.

**Abstract:**

Many variants of urothelial cancer present diagnostic challenges and carry clinical implications that influence prognosis and treatment decisions. The critical issues of treatment-resistant clones are a crucial barrier to care in individuals affected by urothelial carcinoma. Laying the foundations for the resistance evolution, a wide mutational heterogeneity characterizes urothelial carcinoma, noticeable also in patients affected by a early stage disease. In recent years the growing knowledge of the pathogenesis and molecular paths underlying the onset and progression of urothelial cancer are leading to the development of new therapies based on immune checkpoints. Chemotherapy and immunotherapy both operate selectively by shaping the developmental trajectory of urothelial carcinoma in the course of the illness. To date, a promising new therapeutic treatment is represented by antibody-drug conjugates, therapeutic tools that exploit the targeted ability of an antibody to administer cytotoxic drugs directly to the tumor. Indeed, nowadays in the clinical setting there are several treatments available for the treatment of locally advanced or metastatic urothelial cancer, from classic chemotherapeutics such as Gemcitabine, Cisplatin and Carboplatin, Paclitaxel and Docetaxel, to Programmed cell death protein 1 (PD-1) or Programmed death-ligand 1 (PD-L1) inhibitors such as Atezolizumab, Avelumab, Nivolumab, Pembrolizumab, up to anti-nectin 4 Enfortumab Vedotin and Sacituzumab govitecan, which binds Tumor-associated calcium signal transducer 2 (Trop-2) and activates as a topoisomerase inhibitor. The aim of this work is to describe the molecular mechanisms underlying the onset of the urothelial cancer and provide an overview of the immunotherapies that can be used in the clinical setting to counteract it, deepening the efficacy and safety results of the pivotal studies and the place in therapy of these treatments.

## 1. Introduction

### 1.1. Urothelial Cancer

The term “urothelium” is used to refer to the lining epithelium of the urinary tract’s mucosal surfaces, of the pelvis and calyces, of the renal collecting tubules, as well as the ureter, urethra and the bladder. Urothelial carcinoma is associated with high death rate, indeed is globally considered as one of the most malignant carcinomas [1]. Numerous studies show that women have a poorer prognosis; the reasons for the lower survival in women remain not yet fully defined [2,3]. Most bladder tumors are urothelial cancers (UBC). Advanced UBC carries a high rate of poor prognosis, with 5-year relative survival ranging from 4% to 50% [4,5,6]. The commonest urinary system malignancy is the urinary bladder carcinoma. The urothelial carcinoma of the superior tract of the urinary bladder is a rare form of urothelial carcinoma with a poor prognosis. For upper tract urothelial carcinoma and urinary bladder carcinoma, among the main risk factors, evidence shows cigarette smoking and occupational exposure; in fact, some chemical carcinogens substances have been identified as the main cause of most cases of urothelial cancer. Furthermore, recent evidence has shown that genetics plays a key role in the onset and the progression of urothelial cancer [7]. Drug therapy for the treatment of locally advanced or metastatic urothelial carcinoma is making tremendous and rapid progress in recent times [8]. In addition, very different histological variants have recently been identified for urothelial carcinoma (UC). Although platinum-based chemotherapy is the standard treatment, the use of immune checkpoint inhibitors (ICIs) for the treatment of urothelial carcinoma represents an important therapeutic weapon. A very valuable pharmacological strategy is the use of PD-L1/programmed death-ligand 1 (PD-L1) inhibitors, which have been authorized and are indicated for the treatment of adult patients with locally advanced or metastatic urothelial carcinoma. Consequently, tests utilised for PD-L1 in UC, PD-L1 test predictive reliability and the potential of other newly discovered biomarkers are very important. Currently, molecular studies of gene expression profile have become increasingly important for making diagnosis or predicting response to drug treatment. The role of surgery in treating patients with metastatic urothelial carcinoma of the bladder is controversial [9]. The aim of this review is to illustrate the molecular mechanisms and the risk factors underlying the onset of the UC, describing the immunotherapies and the recent developed antibodies as tools available in the clinical practice, deepening the place in therapy of these treatments and the efficacy and safety results of the pivotal studies: the main features are shown in Table 1.

### 1.2. Risks Factors

Several evidences have shown that urothelial forms such as Upper Tract Urothelial Carcinoma (UTUC) and urothelial bladder cancer (UBC) have different risk factors. For example, tobacco smoking is a significant risk factor for UTUC; in fact, evidence reports that the relative risk of developing UTUC among smokers is 2.5 to 7 times higher than that of nonsmokers [10,11,12]. The carcinogenic compounds in cigarettes which can cause bladder cancer have not been definitely detected. Cigarettes contain over 60 carcinogens and reactive oxygen species, which can induce changes in the DNA damage response mechanism, potentially additively or synergistically harming the host response to carcinogenic agents [13]. A prospective analysis of the National Institutes of Health-ARP Diet and Health Study Cohort describes the relation among bladder cancer risk and smoking [14]. This database encloses over 465,000 people treated in the United States, from 1995 to 2006. There has been a meaningful raised risk of bladder cancer onset for both women and men who are current smokers (multivariate adjusted HRs 4.65 and 3.89, respectively). Even though former smokers have showed a reduction of the risk of bladder cancer onset, the risk rested significantly high (HR 2.52 and 2.14 for women and men, respectively). From a meta-analysis study including 88 different studies emerged that for former smokers, current smokers and all smokers people the relative bladder cancer risk values, compared with nonsmokers individuals, had a value of 2.07 (95% CI 1.84–2.33), 3.49 (95% CI 3.13–3.88) and 2.62 (95% CI 2.43–2.83), respectively [15]. Smoking extent seems to be connected to bladder carcinoma aggressiveness. A study conducted over a period of 22 years showed that in 740 patients, heavy smokers (≥30 pack-years) were at risk of developing high-grade cancer and muscle-invasive disease at initial presentation, compared to non-smokers [16]. Recent evidence demonstrated how a dietary exposure to a carcinogen substance contained in Aristolochia plants (aristolochicacid) may be responsible for the development of urothelial tumors of the renal pelvis and ureter [17]. Nephropathy induced by Chinese herbs may be a cause of urothelial carcinoma of the renal pelvis [18]. Occupational exposure has been linked to an increased risk of urothelial bladder cancer. Aromatic amines, to which one is exposed in the chemical industry, are the main carcinogens responsible for an increased risk of developing urothelial cancer [19]. Finally, many epidemiological studies demonstrate the importance of genetic predisposition as a risk factor for the development of UBC and UTUC. In fact, Lynch syndrome patients show anomalies in DNA mismatch repair which are associated with urothelial carcinomas, in particular of the renal pelvis and the ureter [20].

### 1.3. Precision Oncology in Urothelial Cancer

Precision oncology drug therapy, is behind the significant and innovative advances in the treatment of all types of neoplastic diseases leading to profound clinical benefits for patients. The growing knowledge about mutations oncology have supported the development of targeted drugs that interfere with the biological and molecular pathways of neoplastic pathology. In the field of urothelial cancer, precision cancer drug therapy is also making tremendous progress, from molecularly targeted treatments for driver mutations, to molecular biomarkers of treatment responsiveness to new approaches targeting cancer-specific proteins for enhanced tumor killing. The use of personalized therapeutic strategies seems particularly appropriate in urothelial cancer, a tumor characterized by heterogeneity and high mutational burden.

## 2. Immunotherapy

The drug therapy for the urothelial carcinoma (UC) has been marked by the spread of the monoclonal antibodies, which act as immune check point inhibitors. Cancer cells are capable to activate various immune check point pathways, producing a reduction in the immune response by the immune cells [21]. The medicines which block this activation work as a tumor suppressing. A marker widely used as a target is PD-1 and its ligands, PD-L1 and PD-L2: in a physiological situation, their interaction is necessary to maintain haemostasis and to prevent an autoimmune response [22]. However, PD-L1 is present on the surface of some tumor cells which can trigger the link between the circulating PD and PD-L1, silencing the immune response of T cells towards them [23,24]. There are four monoclonal antibodies available for the treatment of the UC, which act by inhibiting the link between PD and PD-L1: specifically, Atezolizumab and Avelumab act by the bond to PD-L1, whereas Nivolumab and Pembrolizumab binding to PD-1. All these medicinal products have been authorized in the United States by the Food and Drug Administration (FDA) and in the European Union by the European Medicine Agency (EMA) for the use in the treatment of UC.

### 2.1. Atezolizumab

Atezolizumab is indicated in the treatment of UC in the second line after failure of platinum-containing chemotherapy or in the first line in patients who are not eligible for platinum therapy and whose tumors have a PD-L1 expression of 5%. The studies that led to the authorization of the indication are three: IMvigor211, IMvigor210 and IMvigor130. All the studies assessed the efficacy and the safety of Atezolizumab.

### 2.2. IMvigor211

931 patients have been enrolled [25]. The primary endpoint considered was the Overall Survival (OS), which was assessed in the Intention To Treat (ITT) population and in subgroups based on the expression of PD-L1 (≥5% and ≥1%); no statistically significant difference has been found between the two arms (*p* = 0.45), it will instead be found in a follow up where the percentage of patients alive after 24 months and after 30 months is significantly higher in the group treated with Atezolizumab compared to the group treated with chemotherapy [26]. Grade 1 and 2 adverse events occurred in more than 10% of patients in both arms, whereas grade 3 and 4 adverse events were less common in patients treated with Atezolizumab than in those treated with chemotherapy (19.8% and 42.7% in the ITT population).

### 2.3. IMvigor130

According to RECIST v1.1 methodology (Response Evaluation Criteria In Solid Tumors), the coprimary endpoints were Progression Free Survival (PFS) and OS. At a mean of 11.8 months of follow-up, PFS was statically superior (*p* = 0.007) in group A versus group C, with a value of 8.2 and 6.3 months, respectively. OS was statistically higher (*p* = 0.027) in group A than in group C, with a value of 16 and 13.4 months, respectively (Table 1). Adverse events leading to the treatment discontinuation occurred in 34% of patients in group A, 6% of patients in group B and 7% of patients in group C [27].

### 2.4. IMvigor210

The study includes two different cohorts of patients. In the first one, according to RECIST v1.1, the primary endpoint was the confirmed Objective Response Rate (ORR). At a follow-up of 17.2 months, ORR occurred in 23% of 119 patients and a complete response emerged in 9%. A subgroup analysis was performed based on the three PD-L1 expression value <1%, between 1 and 5%, >5%, which gave an ORR of 21%, 21% and 28% respectively. In nine patients there were side effects that caused a discontinuation of the treatment, whereas in one case there was death related to the treatment [28]. In the second cohort, 310 patients who experienced disease progression after first-line platinum-based drugs were included. Co-primary efficacy endpoints were the ORR by RECIST and the immune modified RECIST A, applied to assess whether drug efficacy is greater than a 10% historical ORR cutoff. For both endpoints, Atezolizumab was statistically superior to historical control by 10%, regardless of the percentage of PD-L1 expression. In reference to the patient subgroups based on PD-L1 expression, confirmed ORR at 21.1 months has been found in 28% of patients with PD-L1 expression ≥5%, while a value of 19.3% has been found in patients with PD-L1 expression of PD-L1 ≥1%. The 69% of patients had at least one treatment-related adverse event (mostly fatigue and nausea), whereas the 16% of patients had a grade 3–4 event such as fatigue and decreased appetite [29].

### 2.5. Avelumab

Avelumab is indicated both for monotherapy of adult patients with locally advanced or metastatic urothelial carcinoma and for the maintenance of non-progressing platinum-based chemotherapy. The efficacy and the safety of Avelumab were assessed in the B9991001 pivotal study, a phase 3 open label study which involved 700 patients (Table 1) [30]. The primary endpoint considered was OS, that was assessed both in the total population and in patients who expressed PD-L1 positive. At one year of treatment, respectively the 71.3% and the 58.4% of patients treated with Avelumab and with the best supportive care (BSC) were still alive (*p* = 0.001). In reference to the PD-L1 positive subgroup, the value of patients’ OS who have been treated with Avelumab at one year was 79.1%, differently from patients enrolled in the control arm who expressed a value of 60.4% (*p* < 0.001). Treatment-related adverse events of any kind occurred in 77.3% of cases; specifically, grade 3–4 adverse events occurred in 16.6% of cases, and were represented by infusion-related reaction, amylase increase, lipase increase.

### 2.6. Nivolumab

Nivolumab is authorized as monotherapy for adults’ patients after failure of previous platinum-based therapy both for the treatment of locally advanced unresectable or metastatic UC and also for the adjuvant treatment of adult patients with invasive muscle UC with tumor expression of PD -L ≥ 1%, at high risk of relapse after radical resection of the tumor. The studies that led to the authorization of the indication are three: CA209275, CA209274, CA209032. All the studies assessed the efficacy and the safety of Nivolumab.

### 2.7. CA209275

In this pivotal study were enrolled 270 patients. The primary efficacy endpoint which has been assessed was the objective response (OR) confirmed in all treated patients who expressed a tumor PD-L1 level of ≥5% and ≥1%. At a minimum of 6 months follow up, respectively 23 patients out of 81 with a PD-L1 expression ≥5% had a confirmed OR, 29 patients out of 122 with a PD-L1 expression ≥1% had an OR confirmed, 23 patients out of 143 with a PD-L1 <1% had an OR confirmed. Both in total treated patients and in subgroups for PD-L1 expression, the response was better than the historical control established at 10% of patients. Focusing on safety, the 46% of patients had at least one grade 1–2 adverse event (mostly fatigue and skin and endocrine system reactions), whereas the 16% of patients had grade 3–4 adverse events (mostly fatigue diarrhea, gastrointestinal and hepatic events) [31].

### 2.8. CA209032

The primary endpoint of the study is the ORR. At a minimum follow-up of 9 months, ORR was achieved in 19 out of 78 patients who were treated. In 17 patients out of 78, treatment-related adverse events of grade 3–4 occurred, mostly represented from elevated levels of amylase and lipase; two patients had to discontinue the treatment due to adverse events and subsequently died [32]. At a minimum follow-up of 38.8 months, confirmed ORR occurred in 25.6% of patients [33].

### 2.9. CA209274

The primary endpoint was the disease free survival (DFS) both in the total patient population (709 patients) and in the subgroup with PD-L1 ≥1% [34]. At a mean follow-up of approximately 20 months, the median DFS was 20.8 months in the Nivolumab group and 10.8 months in the placebo group in the total sample, with a 6-month percentage of disease free patients equal to 74. 9 % and 60.3%, respectively (*p* < 0.001). On the other hand, in the subgroup of patients with PD-L1 ≥1%, the percentage of disease free patients at 6 months was 74.5% in the nivolumab group and 55.7% in the placebo group (*p* < 0.001). Grade 3–4 treatment-related adverse events occurred in 17.9% of patients on nivolumab (increased lipase and amylase mainly, then diarrhea and colitis) and in 7.2% of patients on placebo. Two patients died from pneumonitis in the nivolumab group.

### 2.10. Pembrolizumab

Pembrolizumab is indicated as monotherapy in adults both for patients with metastatic or locally advanced UC who have previously received a platinum therapy and for patients with metastatic or locally advanced UC whose tumor expresses PD-L1 with a combined positive score (CPS) ≥10%, who are not eligible for a cisplatin chemotherapy. The studies that led to the authorization of the indication are three: KEYNOTE-045, KEYNOTE-052, KEYNOTE-361. KEYNOTE 0–52 and KEYNOTE-361 assessed either the efficacy or the safety of Pembrolizumab, whereas the study KEYNOTE-045 evaluated only the efficacy endpoints.

### 2.11. KEYNOTE-045

This is a phase 3, open label study, where patients were randomized into 2 groups, the pembrolizumab arm and the chemotherapy arm [35]. Co-primary endpoints were OS and PFS, which were assessed in both the total sample and the subgroup of patients expressing a PD-L1 ≥10%. In the total sample of 542 patients, the OS value corresponded to 10.3 months in the pembrolizumab arm and to 7.4 months in the chemotherapy arm (*p* = 0.002), whereas in patients with PD-L1 expression ≥10% the OS value was respectively of 8 and 5.2 months (*p* = 0.005). PFS did not show statistically significant differences in the 2 groups, both in the total sample (*p* = 0.42) and in the PD-L1 subgroup ≥10% (*p* = 0.24).

### 2.12. KEYNOTE-052

This is a phase 2, single-arm study, in which has been recruited patients with advanced UC untreated with chemotherapy due to ineligibility to cisplatin [36]. The primal endpoint has been OR, which was assessed at a median follow-up of 20 weeks in 370 patients. The OR has been centrally evaluated in 89 patients out of 370. A major response to pembrolizumab has been associated with a PD-L1-expression cutoff of 10%; the OR has been centrally evaluated in 42 out of 110 patients who had a combined positive score with a value of 10% or more. Overall, 2% of the patients experienced treatment-related adverse events with a grade 3 or 4, in particular fatigue, whereas 1% of the patients experienced muscle weakness, colitis and alkaline phosphatase increase. The 10% of the patients experienced a severe adverse event. The 5% of the patients died from non-treatment-related adverse events, whereas one patient died from treatment-related adverse events.

### 2.13. KEYNOTE-361

This is a phase 3, open label, 3-arm study that have split patients who were administered only pembrolizumab, patients who were administered only chemotherapy and patients receiving pembrolizumab plus chemotherapy [37]. OS and PFS have been the co-primal endpoints and either in OS values or in PFS values there has not been statistically significant difference among patients’ groups who has been administered pembrolizumab plus chemotherapy and only chemotherapy: the authors concluded that it is not recommended, in the first line, to add pembrolizumab to the platinum-based chemotherapy. Treatment-related grade ≥3 adverse events occurred in 87% of patients in the pembrolizumab plus chemotherapy group (most anemia and neutropenia), 63% in the pembrolizumab alone group (most anemia and gastrointestinal symptoms), 82% in the only chemotherapy (mostly anemia and neutropenia).

## 3. Antibody-Drug Conjugates

A promising new weapon in oncotherapy is represented by antibody-drug conjugates (ADC) [38]. ADC pharmacological action is based on the recognition of a cellular antigen by an antibody to administer a cytotoxic drug [38,39]. ADC have a lower incidence of adverse events and better pharmacokinetic and pharmacodynamic properties comparing to chemotherapy, being an example of personalized peptide nanoparticle technology, useful for diseases such as cancer but also as a new diagnostic tool [38,40]. The first marketing authorization for ADC was issued in the United States of America (USA) for the treatment of some haematological malignancies and some solid tumors [41]. Enfortumab vedotin (EV) has been the first ADC to be authorized in the USA in December 2019, indicated for the treatment of locally advanced or metastatic UC in adults who had been previously administered a platinum-containing chemotherapy and a PD-1 or PD-L1 inhibitor [42]. Two years later, in April 2021, the FDA approved a second ADC with the same therapeutic indication, sacituzumab govitecan (SG) [43]. The following year, the EMA also approved the indication of EV for the treatment of urothelial carcinoma [44]. ADC are medicinal products designed to match the antibody selectivity with the anticancer chemotherapy potential. Indeed, ADC are defined as targeted chemotherapy due to the fact that they include an antibody which contains a cytotoxic active ingredient, which is subsequently released into the tumor by a linker. Each of these three components has characteristics which establish the ADC pharmacokinetics and pharmacodynamics [38,39]. The antibody that recognizes the cancerous antigen as a target represents the main part of an ADC: essential characteristics of the antibody are the specificity of the target and the binding affinity with it. The second constituent of ADC is the payload responsible for the cytotoxic activity, or rather the number of cytostatic molecules that an antibody is able to transport, equivalent to the expression of the drug-antibody ratio [45]. The third component is a linker, which prevents drug delivery to an off-target site. ADC are administered intravenously, circulate in the plasma and after the identification they attack the target cell, subsequently being internalized in order to release the cytotoxic payload that leads to the apoptosis of the tumor cell. Of all urogenital carcinomas, ADC showed the best efficacy profile in CU. The targets of the pharmacological action of EV and SG are respectively Nectin-4 and Trop-2 [46].

### 3.1. Enfortumab Vedotin

Enfortumab vedotin targets nectin-4, an adhesion protein found on the surface of urothelial cancer cells. EV consists of a totally human IgG1 kappa antibody, conjugated to the anti-microtubule MMAE antigen by means of a binder that can be split with a protease enzyme [47]. EV activity is expressed by ADC binding to the cells which express nectin-4, to whom follows ADC-nectin-4 complex internalization and MMAE release due to a proteolytic cleavage, which damages microtubules system in the target cell, causing the blockage of the cycle cell and the cytotoxic cell death of the tumor cell by apoptosis. The efficacy and safety of EV have been assessed in the studies EV-201 and EV-301 [48,49].

### 3.2. EV-301

This is a phase 3, multicenter, open-label, randomized study which compared the efficacy of IV with chemotherapy in 608 adult patients with locally advanced or metastatic CU who had previously been treated with platinum-containing chemotherapy and a PD-1 inhibitor/L1. The primary endpoint of the study was OS. Patients who have been treated with EV (*n* = 301) achieved a median survival of 3.9 months longer than patients who have been treated with chemotherapy (*n* = 307). The median OS was 12.9 versus 9.0 months, respectively.

### 3.3. EV-201

EV efficacy was evaluated in a single-arm, multicenter trial which enrolled 125 patients. The major efficacy outcome measures were confirmed ORR, that was respectively obtained in 52% of the patients; the 20% of the patients achieved a complete response and the 31% a partial response. EV safety as monotherapy was assessed for a median duration of 4.7 months. The following adverse reactions mainly occurred in the clinical study: skin disorders (55% of patients), hyperglycaemia (14% of patients), cases of peripheral neuropathy (52% of patients), cases of dry eye (30% of patients). More specifically, the most common grade 3 or 4 treatment-related adverse events were neutropenia (9% of the patients), maculopapular rash (8% of the patients) and fatigue (7% of the patients).

### 3.4. Sacituzumab Govitecan

SG is a humanized antibody which identifies Trop-2. SN is constituted by a topoisomerase I inhibitor molecule named SN-38 which is covalently bound through a linker to the antibody. Scientific evidence suggests that SN identifies Trop-2 expressing tumor cells to be subsequently internalized and to release SN-38 through linker hydrolysis. SN-38 binds topoisomerase I and hinders binding of single strand breaks induced by topoisomerase I [47]. The resulting DNA damage of the target cell leads to apoptosis of the tumor cell. The efficacy and safety of SG were assessed in the TROPHY (IMMU-132–06; NCT03547973) study, a multicenter, open-label, single-arm, randomized study which involved 113 patients affected by CU who have been previously administered a platinum therapy and either PD-1 or PD-L1 inhibitor [50,51].

### 3.5. Trophy

The primary endpoints of the study have been OS and PFS. At a median follow-up of 9.1 months, OS was calculated as 10.9 months (95% CI 9–13.8), the median PFS was 5.4 (95% CI 3.5–6.9). Severe adverse reactions occurred in 44% of patients, specifically: microbial infections (18%), neutropenia (12%), urinary tract infection (6%), acute kidney injury (6%), bacteremia or sepsis (5 %), diarrhea (4%), small intestinal obstruction, venous thromboembolism and anemia (3% each), thrombocytopenia, abdominal pain, pyrexia and pneumonia (2% each). More in detail, treatment-related adverse events with a grade ≥3 have been expressed as neutropenia (35%), leukopenia (18%), anemia (14%), febrile neutropenia and diarrhea (10%); in the 6% of the cases there have been expressed treatment-related adverse events which leaded to the interruption of the therapy.

## 4. Conclusions

It is evident that urothelial carcinoma represents a threat to the health of the global population, and that the risk factors and the elements underlying the onset are manifold. The analysis of the molecular mechanisms underlying the pathophysiology of UC has made it possible to develop new strategies to treat this pathology and to authorize the marketing of several medicines in recent years, with efficacy and safety data to support these findings. Pharmacotherapy appears to be a crucial weapon for the care and the management of patients with UC. Thanks to the ongoing research that is currently underway, in the future the therapeutic armamentarium will be able to expand further: it will therefore be essential for healthcare professionals to better manage pharmaceutical governance, ensuring the access to care, to allow patients to fully adhere to the pharmacological treatment [52,53,54].

## Figures and Tables

**Table 1:** New therapeutic perspectives.

Drug and Study.	Posology	Indication	CT Phase	Design	Arms	*n*	Primary Endpoint	Control Group	PD Cutoff	Results
Atezolizumab IMvigor211	1200 mg every 3 weeks	Second line after Pt or first line in the ineligible patients	III	Open Label	2	931	OS	Chemotherapy (vinflunine or docetaxel or paclitaxel)	≥5% ≥1%	At 30 months 18.1 % Atezolizumab, 9.8% control
(A) Atezolizumab + Cisplatin IMvigor130	1200 mg every 3 weeks + chemotherapy	First line	III	Medley	3	1213	OS, PFS	(B) Atezolizumab alone, (C) Placebo + platinum based chemotherapy	≥5% ≥1%	PFS: A = 8.2 months, C = 6.3 months OS: A = 16 months, C = 13.4 months
Atezolizumab IMvigor210 cohort 1	1200 mg every 3 weeks	First line	II	-	1	119	ORR	-	≥5% ≥1%	At 17.2 months of follow up ORR in the 23% of patients
Atezolizumab IMvigor210 cohort 2	1200 mg every 3 weeks until progression	Second line after Pt	II	-	1	310	ORR and immune modified RECIST	Historical control ORR = 10%	≥5% ≥1%	At 21.1 months ORR = 28% in PD-L1 ≥ 5% and 19.3% in PD-L1 ≥ 1%
Avelumab B9991001	At a dosage of 10 mg/kg of body weight every 2 weeks	Maintenance of Platinum-based chemotherapy	III	Open Label	2	700	OS	BSC	PD-L1 positive	At 12 months OS Avelumab = 71.3%, OS BSC = 58.4%
Nivolumab CA209275	3 mg/kg	Progressive during or after platinum therapy	II	-	1	270	ORR	Historical control ORR = 10%	≥5% ≥1%	At a minimum follow-up of 6 months ORR: PD-L1 ≥ 5 28%, PD-L1 ≥ 1% 23.8%, PD-L1 < 1% 16%
Nivolumab CA209032	3 mg/kg every 2 weeks until progression	Second line	I/II	-	1	78	ORR	-	≥1%	At a minimum follow-up of 9 months ORR in 19 patients out of 78
Nivolumab CA209274	3 mg/kg	After surgical resection	III	Double Blind	2	709	DFS	Placebo	≥1%	20.8 months Nivolumab, 10.8 placebo
Pembrolizumab KEYNOTE-045	200 mg every 3 weeks or chemotherapy	Second line after Pt	III	Open Label	2	542	OS, PFS	Chemotherapy (vinflunine or docetaxel or paclitaxel)	≥10%	OS in all population 10.3 months in Pembrolizumab, 7.4 months in control arm OS in PD-L1 ≥ 10% respectively 8 e 5.2 months.
Pembrolizumab KEYNOTE-052	200 mg every 3 weeks	First line	II	-	1	374	OR	-	≥10%	89 (24%, 95% CI 20–29) of 370 patients had a centrally assessed OR
(A) Pembrolizumab + chemotherapy KEYNOTE-361	200 mg every 3 weeks or 200 mg every 3 weeks+chemotherapy or chemotherapy alone	First line	III	Open Label	3	1010	OS, PFS	(B) Pembrolizumab alone, (C) Chemotherapy alone	PD-L1 CPS of at least 10	There were no statistically significant differences
Enfortumab + chemotherapy EV-301	1.25 mg/kg on days 1, 8, and 15 of every 28-day cycle	After previous treatments with platinum-containing chemotherapy and a PD-1 inhibitor/L1	III	Open Label	2	608	OS, PFS	Chemotherapy alone	-	OS was longer in the EV group than in the chemotherapy group (12.88 vs. 8.97 months; hazard ratio for death, 0.70; 95% confidence interval [CI], 0.56 to 0.89; *p* = 0.001). PFS was also longer in the EV group than in the chemotherapy group (5.55 vs. 3.71 months; hazard ratio for progression or death, 0.62; 95% CI, 0.51 to 0.75; *p* < 0.001)
Enfortumab EV-201	1.25 mg/kg on days 1, 8, and 15 of every 28-day cycle	After previous treatments with platinum-containing chemotherapy and a PD-1 inhibitor/L1	II	-	1	125	ORR	-	-	ORR: 52% (95% CI 41–62)
Sacituzumab TROPHY-U-01	10 mg/kg on days 1 and 8 of 21-day cycles	After previous treatments who had progressed after prior PLT and CPI	II	Open Label	1	113	ORR, OS, PFS	-	-	DOR: 7.2 months (95% CI, 4.7 to 8.6 months); PFS: 5.4 months (95% CI, 3.5 to 7.2 months); OS: 10.9 months (95% CI, 9.0 to 13.8 months)

## References

[1] Sung H., Ferlay J., Siegel R.L., Laversanne M., Soerjomataram I., Jemal A., Bray F. (2021). Global Cancer Statistics 2020: GLOBOCAN Estimates of Incidence and Mortality Worldwide for 36 Cancers in 185 Countries. CA Cancer, J. Clin..

[2] Dobruch J., Daneshmand S., Fisch M., Lotan Y., Noon A.P., Resnick M.J., Shariat S.F., Zlotta A.R., Boorjian S.A. (2016). Gender and Bladder Cancer: A Collaborative Review of Etiology, Biology, and Outcomes. Eur. Urol..

[3] Andreassen B.K., Grimsrud T.K., Haug E.S. (2018). Bladder cancer survival: Women better off in the long run. Eur. J. Cancer.

[4] Tracey E., Roder D., Luke C., Bishop J. (2009). Bladder cancer survivals in New South Wales, Australia: Why do women have poorer survival than men?. Br. J. Urol..

[5] Garg T., Pinheiro L.C., Atoria C.L., Donat S., Weissman J.S., Herr H.W., Elkin E.B. (2014). Gender Disparities in Hematuria Evaluation and Bladder Cancer Diagnosis: A Population Based Analysis. J. Urol..

[6] Marks P., Soave A., Shariat S.F., Fajkovic H., Fisch M., Rink M. (2016). Female with bladder cancer: What and why is there a difference?. Transl. Androl. Urol..

[7] Miyazaki J., Nishiyama H. (2017). Epidemiology of urothelial carcinoma. Int. J. Urol..

[8] Yuasa T., Urakami S., Yonese J. (2018). Recent advances in medical therapy for metastatic urothelial cancer. Int. J. Clin. Oncol..

[9] Quintens H., Roupret M., Larré S., Neuzillet Y., Pignot G., Compérat E., Wallerand H., Houédé N., Roy C., Soulié M. (2012). Radiochimiothérapie pour le traitement des cancers de vessie infiltrant le muscle: Modalités, surveillance et résultats. Mise Au Point Du Com. De Cancérologie De L’association Française D’urologie.

[10] Colin P., Koenig P., Ouzzane A., Berthon N., Villers A., Biserte J., Roupret M. (2009). Environmental factors involved in car-cinogenesis of urothelial cell carcinomas of the upper urinary tract. BJU Int..

[11] Pommer W., Bronder E., Klimpel A., Helmert U., Greiser E., Molzahn M. (1999). Urothelial cancer at different tumour sites: Role of smoking and habitualintake of analgesics and laxatives. Results of the Berlin Urothelial Cancer Study. Nephrol. Dial. Transplant..

[12] McLaughlin J.K., Silverman D.T., Hsing A.W., Ross R.K., Schoenberg J.B., Yu M.C., Stemhagen A., Lynch C.F., Blot W.J., Fraumeni J.F. (1992). Cigarette smoking and can-cers of the renal pelvis and ureter. Cancer Res..

[13] Borzym-Kluczyk M., Radziejewska I., Zaniewska A., Borzym-Lewszuk A., Szajda S., Knaś M., Zwierz K., Darewicz B. (2009). Effect of smokingon activity of N-acetyl-beta-hexosaminidase in serum and urine of renal can-cer patients. Clin. Biochem..

[14] Freedman N.D., Silverman D.T., Hollenbeck A.R., Schatzkin A., Abnet C.C. (2011). Association between smoking and risk of bladder cancer among men andwomen. JAMA.

[15] Cumberbatch M.G., Rota M., Catto J.W., La Vecchia C. (2016). The role of tobaccosmoke in bladder and kidney carcinogenesis: A com-parison of exposuresand meta-analysis of incidence and mortality risks. Eur. Urol..

[16] Pietzak E.J., Mucksavage P., Guzzo T.J., Malkowicz S.B. (2015). Heavy cigarette smoking and aggressive bladder cancer at initial presen-tation. Urology.

[17] Grollman A.P., Shibutani S., Moriya M., Miller F., Wu L., Moll U., Suzuki N., Fernandes A., Rosenquist T., Medverec Z. (2007). Aristolochic acid and the etiology of endemic (Balkan) nephropathy. Proc. Natl. Acad. Sci. USA.

[18] Nortier J.L., Martinez M.-C.M., Schmeiser H.H., Arlt V.M., Bieler C.A., Petein M., Depierreux M.F., De Pauw L., Abramowicz D., Vereerstraeten P. (2000). Urothelial Carcinoma Associated with the Use of a Chinese Herb (*Aristolochia fangchi*). N. Engl. J. Med..

[19] Naito S., Tanaka K., Koga H., Kotoh S., Hirohata T., Kumazawa J. (1995). Cancer occurrence among dyestuff workers exposed to aro-matic amines. A Long Term Follow-up Study. Cancer.

[20] Gylling A.H., Nieminen T.T., Abdel-Rahman W.M. (2008). Differential cancer pre-disposition in Lynch syndrome: Insights from molec-ular analysis of brain andurinary tract tumors. Carcinogenesis.

[21] Gupta S., Gill D., Poole A., Agarwal N. (2017). Systemic Immunotherapy for Urothelial Cancer: Current Trends and Future Directions. Cancers.

[22] Sun S., Xu L., Zhang X., Pang L., Long Z., Deng C., Zhu J., Zhou S., Wan L., Pang B. (2021). Systematic Assessment of Transcriptomic Biomarkers for Immune Checkpoint Blockade Response in Cancer Immunotherapy. Cancers.

[23] Darvin P., Toor S.M., Sasidharan Nair V., Elkord E. (2018). Immune checkpoint inhibitors: Recent progress and potential biomarkers. Exp. Mol. Med..

[24] Alsaab H.O., Sau S., Alzhrani R., Tatiparti K., Bhise K., Kashaw S.K., Iyer A.K. (2017). PD-1 and PD-L1 Checkpoint Signaling Inhibition for Cancer Immunotherapy: Mechanism, Combinations, and Clinical Outcome. Front. Pharmacol..

[25] Powles T., Durán I., Van Der Heijden M.S., Loriot Y., Vogelzang N.J., De Giorgi U., Oudard S., Retz M.M., Castellano D., Bamias A. (2018). Atezolizumab versus chemotherapy in patients with platinum-treated locally advanced or metastatic urothelial carcinoma (IMvigor211): A multicentre, open-label, phase 3 randomised controlled trial. Lancet.

[26] van der Heijden M.S., Loriot Y., Durán I., Ravaud A., Retz M., Vogelzang N.J., Nelson B., Wang J., Shen X., Powles T. (2021). Atezolizumab Versus Chemotherapy in Patients with Platinum-treated Locally Advanced or Metastatic Urothelial Carcinoma: A Long-term Overall Survival and Safety Update from the Phase 3 IMvigor211 Clinical Trial. Eur. Urol..

[27] Galsky M.D., Arija J.Á.A., Bamias A., Davis I.D., De Santis M., Kikuchi E., Garcia-Del-Muro X., De Giorgi U., Mencinger M., Izumi K. (2020). Atezolizumab with or without chemotherapy in metastatic urothelial cancer (IMvigor130): A multicentre, randomised, placebo-controlled phase 3 trial. Lancet.

[28] Balar A.V., Galsky M.D., Rosenberg J.E., Powles T., Petrylak D.P., Bellmunt J., Loriot Y., Necchi A., Hoffman-Censits J., Perez-Gracia J.L. (2017). Atezolizumab as first-line treatment in cisplatin-ineligible patients with locally advanced and metastatic urothelial carcinoma: A single-arm, multicentre, phase 2 trial. Lancet.

[29] Rosenberg J.E., Hoffman-Censits J., Powles T., van der Heijden M.S., Balar A.V., Necchi A., Dawson N., O’Donnell P.H., Balmanoukian A., Loriot Y. (2016). Atezolizumab in patients with locally advanced and metastatic urothelial carcinoma who have progressed following treatment with platinum-based chemotherapy: A single-arm, multicentre, phase 2 trial. Lancet.

[30] Powles T., Park S.H., Voog E., Caserta C., Valderrama B.P., Gurney H., Kalofonos H., Radulović S., Demey W., Ullén A. (2020). Avelumab Maintenance Therapy for Advanced or Metastatic Urothelial Carcinoma. N. Engl. J. Med..

[31] Sharma P., Retz M., Siefker-Radtke A., Baron A., Necchi A., Bedke J., Plimack E.R., Vaena D., Grimm M.-O., Bracarda S. (2017). Nivolumab in metastatic urothelial carcinoma after platinum therapy (CheckMate 275): A multicentre, single-arm, phase 2 trial. Lancet Oncol..

[32] Sharma P., Callahan M.K., Bono P., Kim J., Spiliopoulou P., Calvo E., Pillai R.N.A., Ott P., de Braud F., Morse M. (2016). Nivolumab monotherapy in recurrent metastatic urothelial carcinoma (CheckMate 032): A multicentre, open-label, two-stage, multi-arm, phase 1/2 trial. Lancet Oncol..

[33] Sharma P., Siefker-Radtke A., De Braud F., Basso U., Calvo E., Bono P., Morse M.A., Ascierto P.A., Lopez-Martin J., Brossart P. (2019). Nivolumab Alone and With Ipilimumab in Previously Treated Metastatic Urothelial Carcinoma: CheckMate 032 Nivolumab 1 mg/kg Plus Ipilimumab 3 mg/kg Expansion Cohort Results. J. Clin. Oncol..

[34] Bajorin D.F., Witjes J.A., Gschwend J.E., Schenker M., Valderrama B.P., Tomita Y., Bamias A., Lebret T., Shariat S.F., Park S.H. (2021). Adjuvant Nivolumab versus Placebo in Muscle-Invasive Urothelial Carcinoma. N. Engl. J. Med..

[35] Bellmunt J., De Wit R., Vaughn D.J., Fradet Y., Lee J.-L., Fong L., Vogelzang N.J., Climent M.A., Petrylak D.P., Choueiri T.K. (2017). Pembrolizumab as Second-Line Therapy for Advanced Urothelial Carcinoma. N. Engl. J. Med..

[36] Balar A.V., Castellano D., O’Donnell P.H., Grivas P., Vuky J., Powles T., Plimack E.R., Hahn N.M., de Wit R., Pang L. (2017). First-line pembrolizumab in cisplatin-ineligible patients with locally advanced and unresectable or metastatic urothelial cancer (KEYNOTE-052): A multicentre, single-arm, phase 2 study. Lancet Oncol..

[37] Powles T., Csőszi T., Özgüroğlu M., Matsubara N., Géczi L., Cheng S.Y.-S., Fradet Y., Oudard S., Vulsteke C., Barrera R.M. (2021). Pembrolizumab alone or combined with chemotherapy versus chemotherapy as first-line therapy for advanced urothelial carcinoma (KEYNOTE-361): A randomised, open-label, phase 3 trial. Lancet Oncol..

[38] Mollica V., Rizzo A., Montironi R., Cheng L., Giunchi F., Schiavina R., Santoni M., Fiorentino M., Lopez-Beltran A., Brunocilla E. (2020). Current Strategies and Novel Therapeutic Approaches for Metastatic Urothelial Carcinoma. Cancers.

[39] Lattanzi M., Rosenberg J.E. (2020). The emerging role of antibody-drug conjugates in urothelial carcinoma. Expert Rev. Anticancer Ther..

[40] Jeong W.J., Bu J., Kubiatowicz L.J., Chen S.S., Kim Y.S., Hong S. (2018). Peptide–nanoparticle conjugates: A next generation of diagnostic and therapeutic platforms?. Nano Converg..

[41] Tong J.T.W., Harris P.W.R., Brimble M.A., Kavianinia I. (2021). An Insight into FDA Approved Antibody-Drug Conjugates for Cancer Therapy. Molecules.

[42] Food and Drug Administration. https://www.fda.gov/drugs/resources-information-approved-drugs/fda-grants-regular-approval-enfortumab-vedotin-ejfv-locally-advanced-or-metastatic-urothelial-cancer.

[43] Food and Drug Administration. https://www.fda.gov/drugs/resources-information-approved-drugs/fda-grants-regular-approval-sacituzumab-govitecan-triple-negative-breast-cancer.

[44] European Medicines Agency. https://www.ema.europa.eu/en/human-regulatory/marketing-authorisation.

[45] Tang Y., Tang F., Yang Y., Zhao L., Zhou H., Dong J. (2017). Realtime analysis on drug-antibody ratio of antibody–drug conjugates for synthesis, process optimization, and quality control. Sci. Rep..

[46] Sigorski D., Różanowski P., Iżycka-Świeszewska E., Wiktorska K. (2022). Antibody–Drug Conjugates in Uro-Oncology. Target. Oncol..

[47] Sarfaty M., Rosenberg J.E. (2020). Antibody-Drug Conjugates in Urothelial Carcinomas. Curr. Oncol. Rep..

[48] Yu E.Y., Petrylak D.P., O’Donnell P.H., Lee J.-L., van der Heijden M.S., Loriot Y., Stein M.N., Necchi A., Kojima T., Harrison M.R. (2021). Enfortumab vedotin after PD-1 or PD-L1 inhibitors in cisplatin-ineligible patients with advanced urothelial carcinoma (EV-201): A multicentre, single-arm, phase 2 trial. Lancet Oncol..

[49] Powles T., Rosenberg J.E., Sonpavde G.P., Loriot Y., Durán I., Lee J.-L., Matsubara N., Vulsteke C., Castellano D., Wu C. (2021). Enfortumab Vedotin in Previously Treated Advanced Urothelial Carcinoma. N. Engl. J. Med..

[50] Tagawa S.T., Balar A.V., Petrylak D.P., Kalebasty A.R., Loriot Y., Fléchon A., Jain R.K., Agarwal N., Bupathi M., Barthelemy P. (2021). TROPHY-U-01: A Phase II Open-Label Study of Sacituzumab Govitecan in Patients with Metastatic Urothelial Carcinoma Progressing After Platinum-Based Chemotherapy and Checkpoint Inhibitors. J. Clin. Oncol..

[51] Ocean A.J., Starodub A.N., Bardia A., Vahdat L.T., Isakoff S.J., Guarino M., Messersmith W.A., Picozzi V.J., Mayer I.A., Wegener W.A. (2017). Sacituzumab govitecan (IMMU-132), an anti-Trop-2-SN-38 antibody-drug conjugate for the treatment of diverse epithelial cancers: Safety and pharmacokinetics. Cancer.

[52] Ferrara F., Santilli P., Vitiello A., Forte G., D’Aiuto V. (2021). Logistics management provides greater efficiency, governance and compliance. Int. J. Clin. Pharm..

[53] Zovi A., Musazzi U.M., D’Angelo C., Piacenza M., Vimercati S., Cilurzo F. (2021). Medicines shortages and the perception of *Healthc.* professionals working in hospitals: An Italian case study. J. Interprofessional Educ. Pr..

[54] Ferrara F., Nava L., Trama U., Nava E., Vitiello A. (2022). The Slow Path to Therapeutic Adherence. Hosp. Pharm..

